# Cellulase activity of a novel bacterial strain *Arthrobacter woluwensis* TDS9: its application on bioconversion of paper mill sludge

**DOI:** 10.1186/s43141-022-00373-w

**Published:** 2022-06-16

**Authors:** Tuhin Das, Ferdausi Ali, Md. Shafiqur Rahman

**Affiliations:** 1 Department of Microbiology, University of Chittagong, Chattogram, 4331 Bangladesh; 2 Department of Biology, Lakehead University, 955 Oliver Road, Thunder Bay, Ontario Canada

**Keywords:** Negative-cost biomass, Bioconversion, Paper mill sludge, Cellulase, Biological treatment

## Abstract

**Background:**

Lignocellulosic biomasses produced from agriculture and forest-based industries are the cheapest or negative-cost biomass with a great potential for biotransformation to value-added bioproducts. Paper mill sludge, an important lignocellulosic biomass creates an environmental threat, which requires financial input for disposal. Thus, this study was aimed to isolate a novel bacterial strain capable of degrading cellulosic biomass including paper mill sludge to produce reducing sugar and other value-added bioproducts.

**Results:**

A novel bacterial strain *Arthrobacter woluwensis* TDS9 isolated from the soil was screened for its cellulolytic activity using carboxymethyl cellulose (CMC) as the sole carbon source. The incubation period, temperature, pH, carbon, and nitrogen sources are the most important factors ruling the CMCase and sugar productions of the strain *A. woluwensis* TDS9, and an alkaline pH (pH 8.0) led to enhanced sugar production up to 1100.09 μg/mL after 72 h of incubation at 25°C in a medium containing 1.5% CMC and 1.25% beef extract. The optimal conditions for maximum CMCase activity were defined, and the potassium ion boosted the CMCase activity up to 1.06 U/mL when the enzymatic reaction was performed for 30 min at 50°C and pH 8 using CMC as a substrate. Moreover, the strain *A. woluwensis* TDS9 produced 433.33 μg/mL reducing sugar from 1% pretreated paper mill sludge. Significant alterations in the structural arrangement of cellulosic fiber of paper mill sludge observed under microscope after each step of chemical treatment process helped for loosening the cellulose fibers and increased the saccharification for enzymatic hydrolysis. Endoglucanase IV (33 KDa) and beta-glucosidase II (53 KDa) were identified in crude enzyme based on the zymogram analysis and substrate specificity.

**Conclusions:**

The research has for the first time proved that this *A. woluwensis* TDS9 strain can efficiently convert cellulose. Therefore, the strain TDS9 could be a potential candidate for cellulase production in an industrial biotransformation process of paper mill sludge to produce reducing sugar. This sugar stream can be further used as a substrate to produce biofuels and other organic acids using another microorganism, which represents a greener alternative to add value to the paper production helping paper mill industries.

## Background

Cellulose, being one of the mostly found biomaterials, is present in plant biomass interlinked with lignin and hemicellulose. The cellulose is a polymeric polysaccharide of 𝛃-1,4-linked 𝛃-glucose molecules, and the degree of polymerization ranges from 10000 to 15000 [1]. In cellulose, each anhydroglucose ring is linked covalently through an oxygen and bonded to the C1 of one glucose to the C4 of the next ring (1→4 linkage) [2].

The enzyme family responsible for the hydrolysis of cellulose is known as cellulase [3–7]. Cellulases are a group of enzymes produced by a wide range of microorganisms including fungi, bacteria, and actinomycetes that catalyze the hydrolysis of cellulose to produce monosaccharides and shorter polysaccharides or oligosaccharides [7–11]. Based on the information found in Carbohydrate-Active Enzymes database (CAZy) [12], cellulase belongs to glycoside hydrolase EC 3.2.1 group of enzymes. Cellulase comprises catalytic and non-catalytic modules. The catalytic modules have been classified into numerous families based on their amino acid sequences and crystal structures. The non-catalytic carbohydrate-binding modules and/or other domains located at N- or C-terminus of a catalytic module.

Cellulolysis is a complex process involving a group of enzymes belonging to cellulase family and multiple steps of enzymatic hydrolysis in the presence of water [13–16]. The complete hydrolysis of cellulose polymer into glucose residues involves at least three types of enzymes in synergy [17]. The endoglucanase attacks amorphous region of cellulose at random site and cleave the internal bonds of the glycan chains providing reducing and/or nonreducing ends of cellooligosaccharides for cellobiohydrolases; therefore, cellobiohydrolases hydrolyse those short chains and yielding cellobiose as a major product. The glucosidase finally hydrolyses cellobiose to produce glucose.

The booming of papermill industries all over the world has led to generate a large amount of primary and secondary sludges, created an environment friendly disposal issue. It is estimated that paper mills produce 300–350 million tons sludges per year, which ensures its availability as the substrate for biofuel production [18]. These paper sludges, unless properly treated pose major threat to the environment as well as to aquatic and agricultural fields [19–21]. So, we need to take eco-symbiotic and economically efficient initiative to relieve the environment from this burden and to achieve a greener environment. Paper mill sludge is the final solid waste recovered from the wastewater treatment process in pulp and paper mills. Sludge discharged from paper mills is divided into four categories: (1) primary sludge (PS) that is the by-product of virgin wood fiber coming from primary screening of wastewater, (2) de-inking paper sludge (DPS) that is the by-product of ink and other dye removal from recycled or waste papers, (3) secondary sludge (SS) that comes from the secondary screening of wastewater treatment system during pulping process, and finally (4) combined primary and secondary sludge that contain a mixture of both primary and secondary sludge characterized with longer fiber length [22]. The primary sludge of paper mill contains a massive amount of lignocellulosic (cellulose, hemicellulose, and lignin) biomass.

Sludge disposal is nowadays a contemporary issue as current disposal methods (landfilling, incineration, and land spreading, etc.) are not economically and environmentally attractive. Over 50% of the total paper mills sludge produced are currently being landfilled or incinerated, which cause both environmental and financial problem [23]. On average, 60% of the total wastewater treatment costs of pulp and paper mills account for sludge disposal [24]. Therefore, paper mill sludge is now considered as a negative-cost biomass. The primary sludge of paper mill contains a significant amount of cellulose, which can be utilized by carboxymethylcellulase (CMCase) producing microorganisms to produce reducing sugar [25]. While conventional waste management methods require significant financial input, bioremediation can play a more efficient role both from economic point of view as well as environmental safety. Consequently, this study was aimed for isolating a potential cellulolytic bacterial candidate which can degrade cellulosic biomass in a cost-effective and eco-friendly way.

Bioconversion process of lignocellulosic biomass consists of various types of processes including physical, chemical, and biological processes [26]. However, each of these processes have their own drawbacks when implemented on a large scale [27, 28]. One of the major hurdles of biomass conversion into biofuel is the high cost of catalytic enzymes and the lack of their reusability and stability. The nanotechnology comes as a boon with its ability to stabilize the biocatalysts and immobilize the enzymes on nanostructures for better catalytic efficiency [29–31]. Nanotechnology has achieved these qualities in two main methods: covalent bonding of different enzymes on nanomaterials and employing functionalized nanoparticles [32, 33]. Nanoparticles are also well known for their bioremedial potential as well as photocatalytic activity to degrade environmental pollutants [34–36]. However, the high cost of processing hinders the effectiveness of the current nanotechnology technique employed on the conversion of lignocellulosic biomasses. Therefore, our study creates an opportunity to valorize the lignocellulosic biomasses including paper mill sludge through a cost-effective biological recycling process that can increase the efficiency of enzymatic hydrolysis.

Even though there are a number of cellulase producing microbial strains commercially available, most of them are not efficient enough to utilize paper mill sludge. Paper sludge contains fair amount of harmful chemicals including acids, alkali, and bleaches that inhibit the growth of microorganisms. Furthermore, the structural arrangement of paper mill sludge is not favorable for microbial degradation due to a complex association of hemicellulose and xylan with cellulosic fibres. Hence, the chemical treatment is required prior to bioconversion of such complex biopolymers. The aim of this research work is the enzymatic degradation of paper mill sludge using efficient cellulase producing bacterial strain to produce sugar; therefore, this bioconversion process was designed to removal of harmful chemical residues from the paper mill sludge by chemical treatment to make cellulose fibres available for enzymatic hydrolysis of cellulase producing microorganism. The uniqueness of our research from other existing study on the conversion of paper mill sludge is that it comprises both chemical treatment and biological agent for successful conversion of paper mill sludge; as no other study attempted the same as far as we know. This sugar residue can be further converted to value-added chemicals. In this research work, a novel bacterial strain *Arthrobacter woluwensis* TDS9 was isolated and characterized, which can efficiently produce CMCase for utilizing cellulosic substrate from lignocellulosic biomass paper mill sludge to produce sugar streams. Therefore, this research might have a significant contribution to the development of biorefinery and paper mill industries.

## Methods

### Isolation of bacterial strain

The soil sample for isolation of paper mill waste degrading and cellulase producing bacteria was collected from degraded paper mill waste of Karnaphuli Paper Mill located at Chandraghona (Latitude: 22.4796389, Longitude: 92.133864), Chattogram, Bangladesh.

About 5.0 g of the collected sample was inoculated into 100 mL of minimal salt (MS) medium (0.5 g/L NaNO_3_, 1.0 g/L K_2_HPO_4_, 0.5 g/L KCl, and 0.5 g/L MgSO_4_) supplemented with 3% primary sludge of paper mill as a sole carbon source cellulose in a 250-mL Erlenmeyer flask, incubated at 35°C with shaking (150 rpm) for 5 days. Following incubation, cellulolytic bacterial isolates were isolated from enriched culture broth using spread plate method. Briefly, 100 μL of enriched broth or diluted enriched broth culture was spread on Luria-Bertani (LB) agar (10.0 g/L peptone, 5.0 g/L yeast extract, 5.0 g/L NaCl, and 15 g/L agar) plate using sterilized glass spreader. Plates were incubated at 35°C for 24h. Bacterial colonies were selected from these plates based on their morphological features such as colony morphology, colony color, and colony size. Single colonies with distinctive visual characteristics were picked and streaked on LB agar plates. Following isolation, pure bacterial isolates were screened for their cellulase activities.

### Screening for carboxymethyl cellulase (CMCase) activity

Bacterial isolates were grown in tubes containing 5.0 mL LB broth (10.0 g/L peptone, 5.0 g/L yeast extract, and 5.0 g/L NaCl) for 24h at 35±2^o^C with shaking at 200 rpm. A subsequent incubation of 5.0 μL of culture broth was dropped on the center of CZAPEK’s agar (10.0 g/L CMC, 1.0 g/L K_2_HPO_4_, 0.5 g/L MgSO_4_. 7H_2_O, 0.5 g/L KCl, 2.0 g/L NaNO_3_, 0.01 g/L FeSO_4_, 15.0 g/L agar) plate, incubated at 35°C for 48h. Following incubation, the plates were flooded with 1% Congo red followed by washing with 1M NaCl to see the cellulolytic activity of bacterial isolates. This primary screening was further confirmed by a semi-quantitative method using Gram’s iodine solution (2.0 g potassium iodide, 1.0 g iodine crystal, and 300 mL distilled water). The formation of a clear zone of hydrolysis (clear zone) indicated the cellulose degradation by bacterial CMCase. The diameter of the clear zone was measured in millimeter. Therefore, the largest zone of hydrolysis producing bacterial isolate TDS9 was selected as a potential candidate for utilizing paper mill sludge to produce CMCase as well as reducing sugar.

### Identification of bacterial isolate

The most promising bacterial isolate TDS9 was identified using 16s rRNA gene sequencing. The isolate TDS9 was cultured in LB broth (10.0 g/L peptone, 5.0 g/L yeast extract, and 5.0 g/L NaCl) for 24h at 35°C. Following incubation, the cells from 1000 μL of broth culture were collected by centrifugation at 12,000 rpm. The genomic DNA was isolated from these bacterial cells using DNA extraction kit (Presto™ mini genomic DNA Bacteria kit, Geneaid, USA) following manufacturer protocols. The extracted DNA was amplified by PCR (polymerase chain reaction) using universal 16S primers 27F (5′-AGAGTTTGATCNTGGCTCAG-3′) and 1492R (5′-GCTTACCTTGTTACGACTT-3′). The resulting PCR product was purified using QIAquick PCR Purification Kit (QIAGEN, Germany) according to manufacturer’s protocol. The purified PCR product was sequenced using the Applied Biosystems BigDyeTM Terminators v3.1 (Thermo Fisher Scientific, USA). The sequence similarity analysis was carried out by comparing the 16S rRNA sequences of the selected strains from sequence data in the GenBank database of the National Center for Biotechnology Information (NCBI) using the “blastn” suite optimized for “Highly similar sequences (Megablast)” [37, 38].

Finally, the sequences of 16S rRNA were aligned using the multiple sequence alignment program CLUSTAL. Phylogenetic and molecular evolutionary analyses were processed through the molecular evolutionary genetics analysis software MEGA X using the neighbor-joining method [39–41]. The identification of selected isolate TDS9 was confirmed using morphological, cultural, and biochemical characteristics [42].

### Fermentation medium and culture condition

Bacterial isolate TDS9 was grown in LB broth medium, and 1.0 mL of overnight culture was transferred into 200 mL conical flask containing 50 mL of minimal salt (MS) medium supplemented with CMC and/paper mill sludge as the sole carbon source. When indicated, the MS medium was supplemented with a specified concentration of different nitrogen and carbon sources. The flasks were incubated in a shaker incubator at 200 rpm. Following incubation, the cell-free supernatant was collected from the culture broth by centrifugating at 4°C and 10,000 rpm for 10 min, which was used for estimation of sugar concentration. The release of reducing sugar from CMC or paper mill sludge was determined using the dinitrosalicylic acid (DNS) method [43–45]. Bacterial growth was observed at 24h intervals. Biomass or cell growth was measured as absorbance at 600 nm (OD_600_) using microplate spectrophotometer. The extracellular protein was estimated using the standard method [46, 47].

### Quantitative assay of reducing sugar and CMCase

The released reducing sugar from CMC or paper mill sludge by CMCase enzyme was analyzed using dinitrosalicylic acid (DNS) method. A modified microplate-based assay method was used for measuring reducing sugars. Briefly, 20 μL of cell-free supernatant (crude enzyme) was mixed with 80 μL of 0.5% CMC and 50 mM citrate buffer at pH 6.0, incubated for 30 min at 50°C [48–50]. The reaction was terminated by adding 200 μL DNS solution (3.15 g/L DNS, 10.48 g/L NaOH, 91.0 g/L Na-K tartrate, 2.5 g/L phenol, and 2.5 g/L sodium metabisulfite). The mixture was boiled for 5 min. The absorbance was determined at OD540_nm_ using microplate reader spectrophotometer. All experiments were repeated at least three. The CMCase production was determined by estimating the amount of reducing sugar by DNS method.

### Optimization of culture condition for CMCase production

Microbial growth and metabolite production are significantly influenced by different environmental as well as chemical factors leading to highest enzyme production when they are cultured in their optimum environment. Thus, the effect of incubation period, temperature, pH, carbon, and nitrogen sources on the production of reducing sugar and CMCase were determined.

Cells from slant culture was inoculated into a LB broth medium to prepare the seed culture. After 24 h of incubation at 35°C under aerobic condition, the seed culture was inoculated into fermentation medium for optimization of fermentation parameters. In case of sugar production, batch fermentations were carried out in conical flasks containing a 50-mL fermentation medium (MS broth medium supplemented with CMC) with 100 μl of 24h seed culture. Aerobic condition was maintained by incubating the culture flasks in a shaker incubator at 200 rpm. For optimizing the incubation time for maximum sugar production, the culture flasks were incubated at 35±2°C for different incubation times (24, 48, 72, 96, 120, and 144 h). After optimizing the incubation time for maximum sugar production, the strain TDS9 was cultured at different temperatures (20, 25, 35, 45, and 55°C) for determining the optimum incubation temperature. Likewise, each parameter was optimized by keeping the values constant that were optimized in the preceding stages. Initial pH of the culture medium was also optimized using a range of medium pH from 3 to 9. In addition, 1% of several carbon (glucose, xylose, lactose, cellobiose, starch, fructose, sucrose, salicin, glycerol, CMC, filter paper, cotton, rice straw, and paper sludge) and nitrogen (ammonium sulfate, sodium nitrate, potassium nitrate, ammonium chloride, urea, soya bean meal, yeast extract, tryptone, beef extract, and peptone) sources were tested to optimize the culture media composition for maximum sugar productions. When indicated, the medium was supplemented with a specified concentration of different nitrogen and carbon sources. The pH of the medium was adjusted with 1 M NaOH or 1 M HCl depending on the experiment. The cell-free supernatant was collected by centrifuging the culture broth at 4°C and10,000 rpm for 10 min, used for the estimation sugar concentration. All experiments were performed in triplicate, and the results are expressed as mean values of three replicates. The standard deviation of the results obtained from triplicate experiments were shown as error bars.

### Characterization of CMCase (cellulase) enzyme

For partial purification of the enzyme, 70% ammonium sulfate was used in cell-free culture broth and kept it overnight at 4°C to allow precipitation. The precipitate was collected by centrifuging the solution at 10,000 rpm for 10 min at 4°C. The precipitate was dissolved in 50-mM sodium citrate buffer (pH 6) and used for determining the molecular weight of enzyme by SDS-PAGE analysis and enzyme activity. SDS-PAGE was performed using 12% (w/v) polyacrylamide gel according to the protocol described by Laemmli [51] and Brunelle and Green [52]. The separated enzyme and molecular markers containing gel was stained overnight in Coomassie Brilliant Blue R-250 followed by destaining with the destaining solution for 1–3 h. The approximate molecular weight of the enzyme was determined from the bands developed in the gel using standard protein marker (Puregene genetix brand, pre-stained protein ladder, lot no: 3 color protein ladder pg-pmt 2922, lot: 00139874).

### Factors affecting CMCase activity

The effects of various physico-chemical parameters along with different carbon and nitrogen sources, metal ions, and surfactants on CMCase activity were determined in cell-free supernatant of bacterial culture broth, which was used as crude enzyme solution. The CMCase activity was assayed using the International Union of Pure and Applied Chemistry (IUPAC) guideline [53, 54] which was designed to measure cellulase activity in terms of CMCase units per milliliter of original enzyme solution. One unit (U) of enzyme activity is defined as the amount of enzyme that releases 1.0 μmol of reducing sugars per minute [54, 55]. For the effect of reaction time, temperature and pH on enzyme activity enzyme-substrate reactions were carried out at a wide range reaction time, temperature, and pH.

### Pre-treatment of paper mill sludge

Primary sludge of paper mill was collected from Domtar’s Windsor Mill (Windsor, Canada). The Paper mill sludge contained 66.7% cellulose, 14.4% lignin, 13.8% hemicellulose, and 5.1% ash. The primarily sludge is inaccessible to the microbial attack due to the structural complexity and residual toxic chemical substituent used during pulping process. However, the sludge was washed with distilled water under continuous stirring for 3 h, filtered to remove other impurities, dried overnight in an oven at 55°C, and shredded into smaller pieces. Moreover, the shredded sludge was subjected to chemical treatment process for removing hemicellulose and lignin contents present in association with the cellulosic fiber to make available for microbial degradation. Therefore, the shredded sludge was submerged in 7% NaOH at a ratio of 1:10 (w/v). The process was carried out thrice under continuous mechanical stirring at 80°C for 3 h. Each time, the sludge was washed with distilled water until pH becomes neutral. Subsequently, the sludge was treated with H_2_O_2_ (11% v/v) under continuous mechanical stirring, where the pH was adjusted to 11 using 7% NaOH and this treatment process was performed thrice (3 h at 45°C in each step). Finally, the sludge was washed with distilled water for several times until the pH became neutral, dried again, and shredded into small pieces.

### Microbial degradation of sludge in the presence of inducers

Several complex carbohydrates (cellobiose, lactose, sucrose, CMC, glucose, and fructose) were used in the culture medium at a concentration of 0.15% (w/v) as the inducers [56]. Each of these inducers was incorporated in separate culture flasks with 1% of chemically treated paper mill sludge in previously optimized culture media to assess their activity to induce cellulase production. Overnight grown seed culture was used as an inoculum and incubated in the previously optimized culture condition. After incubation, sugar was estimated from the culture filtrate to determine the cellulase activity.

## Results

### Isolation and screening for CMCase activity

A total of 83 bacterial isolates were isolated based on their colony color, appearance, and characteristics from LB agar plate. Furthermore, the isolates were screened for their CMCase activity. Among the 83 isolates, the isolate TDS9 showed the largest hydrolyzing zones 12.75 and 8.47 mm in diameters on CZAPEK’s agar plates using 1% CMC and 1% pre-treated paper mill sludge, respectively (Table 1). Also, the isolate TDS9 produced 683.33 and 350.00 μg/mL reducing sugar from 1% CMC and 1% pre-treated paper mill sludge, respectively (Table 1).

**Table 1:** Screening for cellulase activities and reducing sugar yields of the strain TDS9

Carbon source	Cellulose hydrolyzing zone (mm)	Reducing sugar (μg/mL)	Enzyme activity (U/mL)
CMC 1%	12.75	683.33	0.253
Paper mill sludge 1%	8.47	350.00	0.13

All the experiments were performed in triplicate, and the mean was presented in the table

### Identification and evolutionary relationship of the isolates

**Fig. 1:**
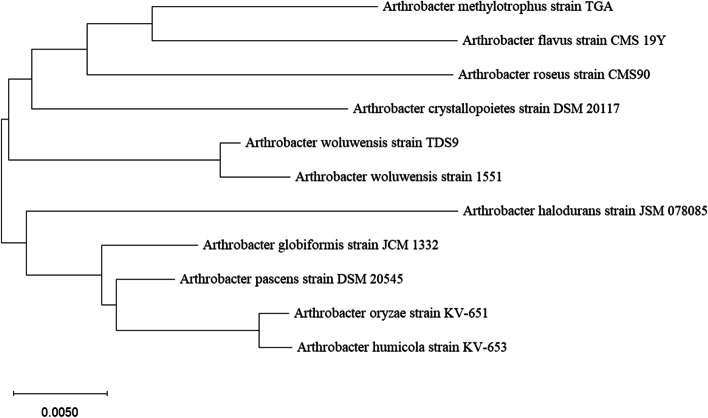
Phylogenetic tree derived by neighbor joining method using 16s rRNA gene sequencing of *A. woluwensis* TDS9 and other species of *Arthrobacter*, showing evolutionary relationship between TDS9 and other related species. Blast information from the NCBI and MEGA software of version 10.0 was used to build the phylogenetic tree

The selected bacterial isolate TDS9 was identified based on 16s rRNA gene sequencing. The sequencing result was submitted into the nucleotide BLAST of the National Center for Biotechnology Information (NCBI) database for possible identification based on sequence similarity. The NCBI BLAST result showed 99.90% similarity of the isolate TDS9 with a previously reported strain A*rthrobacter woluwensis* 1551, and the phylogenetic analysis confirmed its relatedness to the genus *Arthrobacter* (Fig. 1). Furthermore, the 16s rRNA gene sequence of TDS9 has been submitted to the NCBI GenBank for an accession number, and the very strain reported in this paper has been assigned as a new strain *Arthrobacter woluwensis* TDS9 with an accession number MT071300. Moreover, the identification of *Arthrobacter woluwensis* TDS9 strain was confirmed using morphological, cultural, and biochemical properties (Table 2).

**Table 2:** Morphological, cultural, and biochemical characteristics of the bacterial strain TDS9

Characteristics	Identification tests	TDS9
**Morphological studies**	Gram staining	Positive
	Shape	Short rod
	Formation	Single
**Cultural studies**	Colony form	Circular, entire
	Colony elevation	Low convex
	Internal structure of the colony	Translucent
	Colony color	Off-white
	Slant growth	Filiform
	Growth in broth	Sediment
**Biochemical studies**	Oxidase test	Negative
	Catalase test	Positive
	Proteolysis test	Positive
	Indole test	Negative
	Citrate test	Positive
	TSI test	No fermentation
	Methyl red test	Negative
	Voges-Proskauer test	Positive
	Fermentation test:	No fermentation for any sugar
	Monosaccharide-arabinose	
	Xylose	
	Glucose	
	Fructose	
	Disaccharide-sucrose	
	Lactose	
	Polysaccharide-raffinose	
	Starch	
	Sugar alcohol-glycerol	
	Mannitol	
	Casein hydrolysis	Positive
	Starch hydrolysis	Negative

### Optimization of culture conditions

**Fig. 2:**
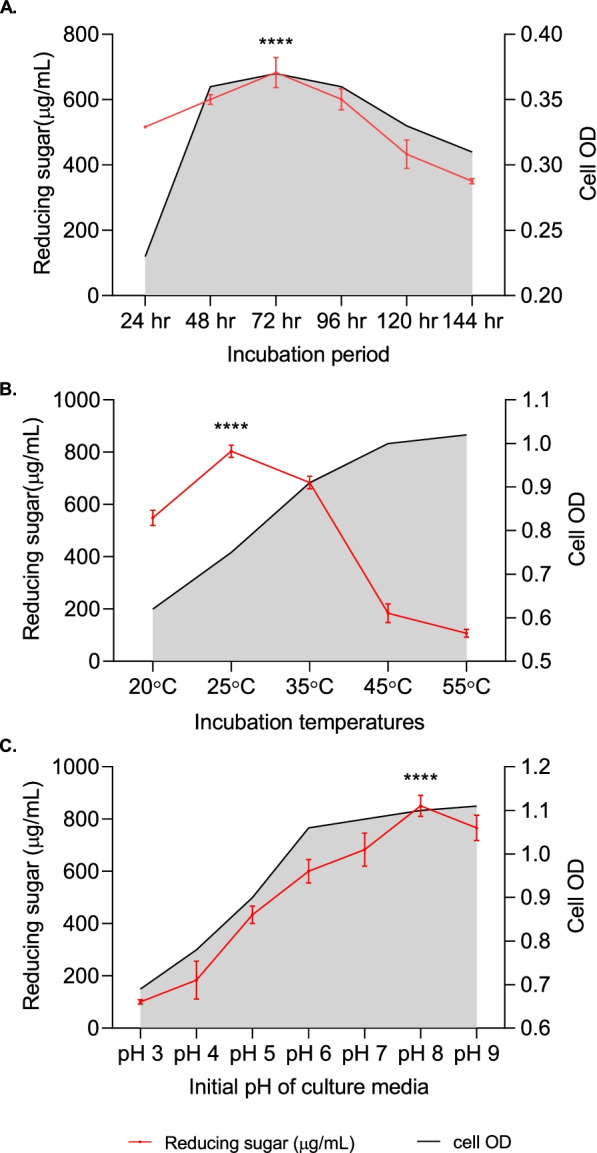
Effect of **A** incubation period, **B** temperature, and **C** initial pH of culture media on the production of reducing sugar by *Arthrobacter* *woluwensis* TDS9. Cell OD was measured at 600nm wavelength. Upon optimization, each culture parameter was kept constant in the following steps. ^****^ indicates statistical significance value, *P* < 0.0001

**Fig. 3:**
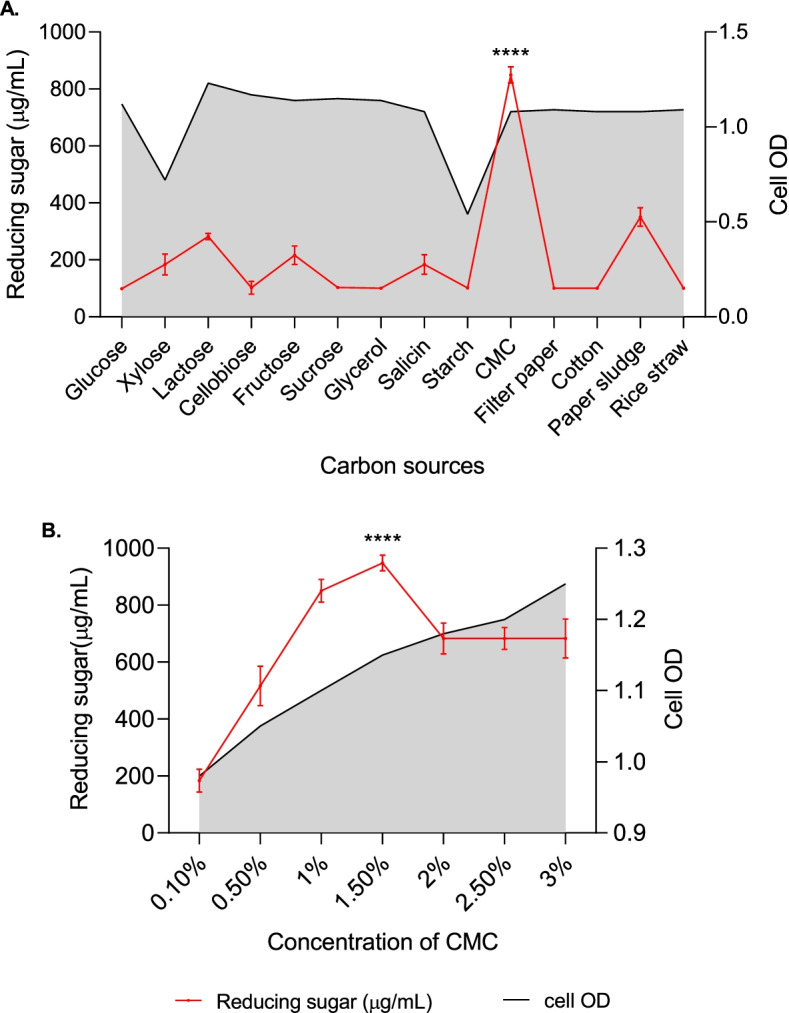
Effect of **A** different carbon sources and **B** different concentrations of CMC on the production of reducing sugar by *Arthrobacter woluwensis* TDS9. Incubation of the culture was performed at 25°C for 72 h maintaining the initial medium pH 8. Cell OD was measured at 600nm wavelength. ^****^ indicates statistical significance value, *P* < 0.0001

**Fig. 4:**
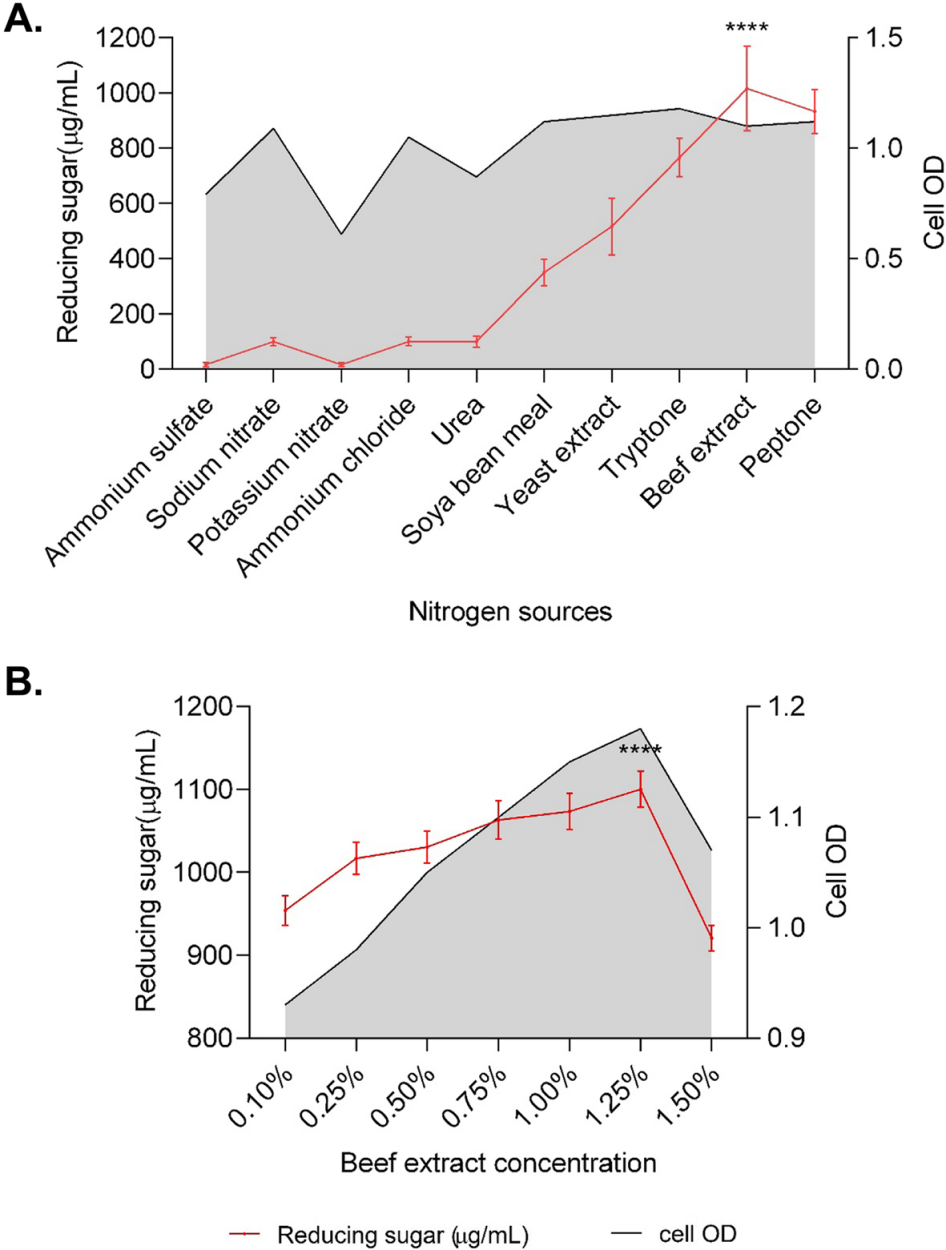
Effect of **A** different nitrogen sources and **B** different concentrations of the beef extract on the production of reducing sugar and extracellular protein by *Arthrobacter woluwensis* TDS9. MS medium with 1.5% CMC as a sole carbon source and medium initial pH was 8. Incubation temperature and incubation time were 25°C and 72 h, respectively. Cell OD was measured at 600nm wavelength. ^****^ indicates statistical significance value, *P* < 0.0001

For maximum production of reducing sugar from MS medium supplemented with CMC (biomass) using the novel strain *A. woluwensis* TDS9, the culture conditions (incubation period, time, temperature, pH, carbon, and nitrogen sources) were optimized, and the results are presented in Figs. 2, 3, and 4. As shown in Fig. 2A, the maximum sugar production 683.00 μg/mL was obtained after 72 h of incubation. The CMCase activity of the strain TDS9 has been significantly influenced by the incubation temperature of growth culture; thus, the optimum incubation temperature for maximum reducing sugar production (803.49 μg/mL) was 25°C (Fig. 2B). Also, the yields of reducing sugar were decreased gradually with the increase of incubation temperature.

However, the initial pH of the culture medium had played a significant role on reducing sugar production by *A. woluwensis* TDS9, and the results are shown in Fig. 2C. Consequently, when *A. woluwensis* TDS9 was cultured in the medium with different initial pH, the maximum amount of reducing sugar (850.24 μg/mL) was attained at pH 8 (Fig. 2C). The concentrations of sugar in culture medium were raised with the increase of medium pH up to 8 and then decreased at pH 9.

The impact of fourteen different carbon sources including CMC on the production of reducing sugar by *A. woluwensis* TDS9 was investigated under optimized temperature (25°C), pH (8), and incubation time (72 h) of the culture, and the results are presented in Fig. 3. As shown in Fig. 3, the results showed that the maximum sugar concentration obtained after 72 h of incubation using *A. woluwensis* TDS9 strain was 947.94 μg/mL when 1.5% CMC was used as a sole carbon source.

Moreover, the effects of different nitrogen sources on the production of reducing sugar by the strain *A. woluwensis* TDS9 are presented in Fig. 4. The strain TDS produced the highest amount (1100.09 μg/mL) of reducing sugar when grown with 1.25% (w/v) beef extract as a nitrogen supplement (Fig. 4).

### SDS-PAGE and zymogram analysis

**Fig. 5:**
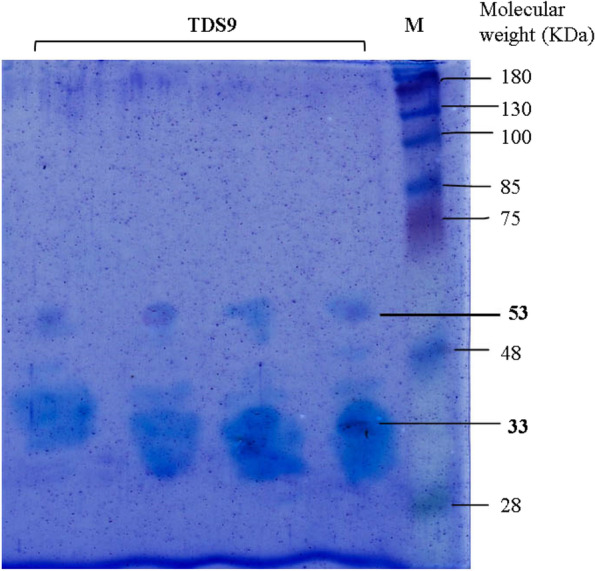
Photograph of SDS-PAGE gel after staining with Coomasie blue stain. The pre-stained protein marker (ladder) is on the right side of the gel with the bands labeled with their molecular weight in KDa. Protein bands of crude enzyme from TDS9 is marked in bold font

Gel picture of SDS-PAGE after staining with Coomassie blue stain revealed that there were two enzyme bands with molecular weight of 33 KDa and 53 KDa, respectively (Fig. 5). According to the “Worthington Enzyme Manual,” the two types of cellulases from *A. woluwensis* were identified as endoglucanase IV (33 KDa) and beta-glucosidase II (53 KDa) based on their molecular weight and substrate (CMC) specificity [57].

### CMCase activity

**Fig. 6:**
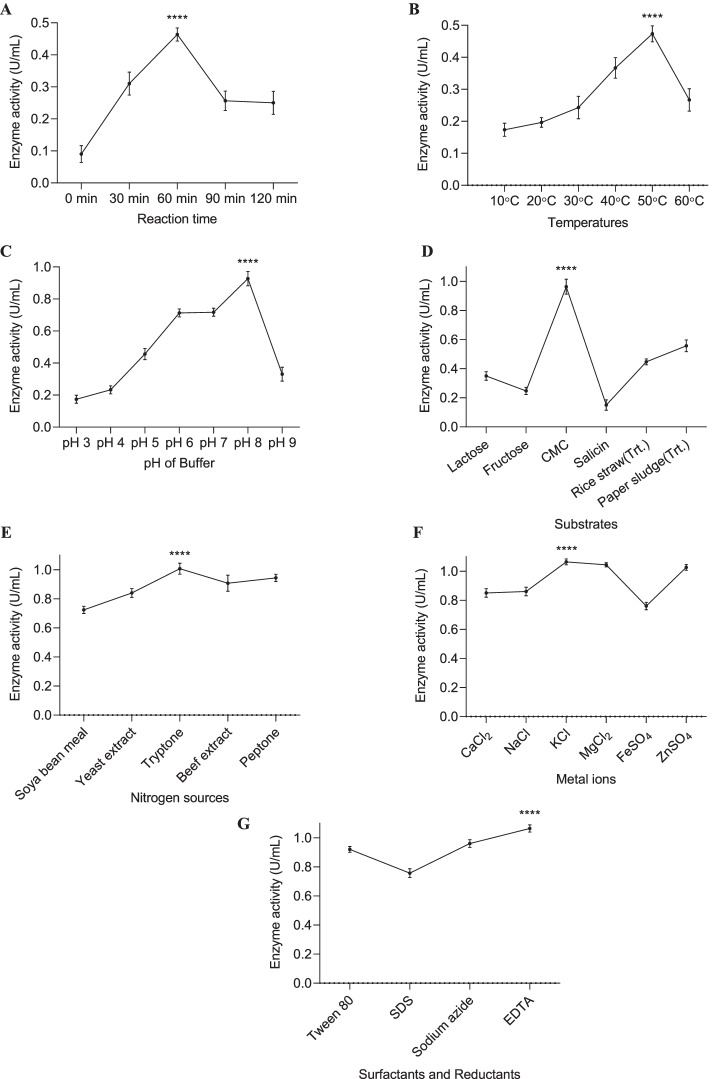
Effect of **A** reaction time, **B** temperature, **C** pH of buffer, **D** substrates, **E** nitrogen sources, **F** metal ions, and **G** surfactants and reductants on enzyme activity obtained from *A. woluwensis* TDS9. Each parameter was kept constant in the following steps upon optimization. ^****^indicates statistical significance value, *P* < 0.0001

The CMCase activity of the newly isolated bacterial strain *A. woluwensis* TDS9 was studied under a wide range of reaction period, temperature, and pH. The optimum enzyme activity (0.46 U/mL) was achieved after 60 min of reaction time (Fig. 6A) at 50°C, indicating the thermal stability of the cellulase for a long period of reaction (Fig. 6B). While assaying the effect of different pH on the CMCase activity of the cellulase from *A. woluwensis* TDS9, the highest enzyme activity (0.93 U/mL) was observed at pH 8 (Fig. 6C). The CMCase activity was decreased at pH 9.

Crude enzyme from *A. woluwensis* TDS9 showed the highest CMCase activity (0.96 U/mL) against carboxymethyl cellulose (CMC) followed by chemically treated paper sludge (0.56 U/mL), which was also our target biomass in this study (Fig. 6D). While studying the effect of different nitrogen sources on the CMCase activity of the cellulase from *A. woluwensis* TDS9, tryptone induced the highest CMCase activity of 1.01 U/mL (Fig. 6E).

The studies on the effect of several metal ions on CMCase activity revealed that the treatment with a 10mM concentration of K^+^, Mg^2+^, and Zn^2+^ enhanced the CMCase activity slightly, while Fe^2+^ inhibited the enzyme activity a bit with the highest being 1.06 U/mL and lowest 0.76 U/mL (Fig. 6F). On the other hand, surfactants and reductants had significant effect on the enzymatic activity of cellulases from *A. woluwensis*. EDTA was found to induce the enzyme activity while SDS significantly inhibited the CMCase activity (Fig. 6G).

### Utilization of paper mill sludge

**Fig. 7:**
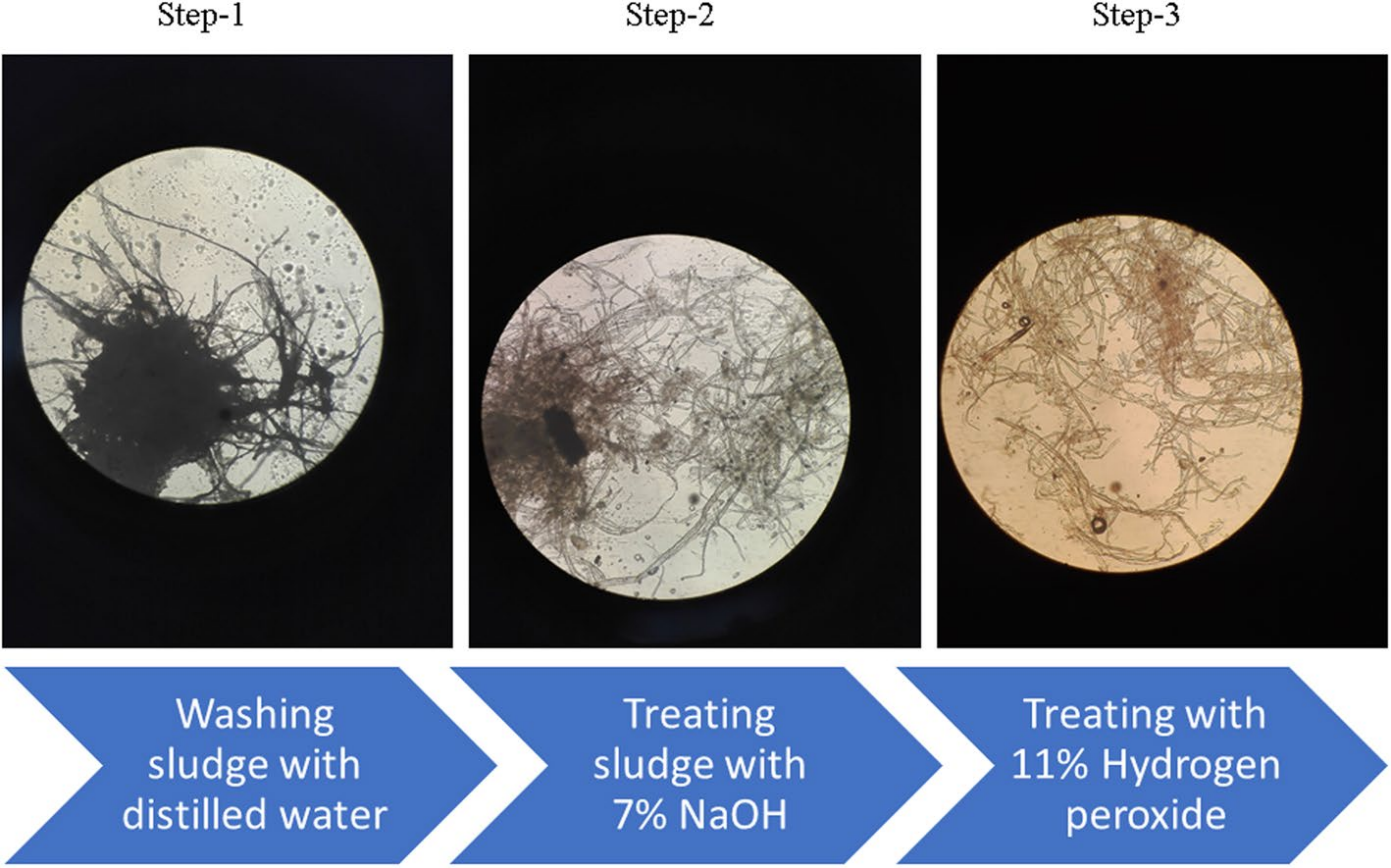
Structural changes of paper sludge throughout chemical treatment process observed under microscope at 10⨯ magnification. Filamentous parts indicate cellulosic fiber sequentially getting loosened and accessible

The chemical treatment process loosened the cellulose fiber from residual lignin and hemicellulose components as observed under microscope at 10⨯ magnification (Fig. 7). A small amount of simple and synthetic carbon source aided microbial growth and metabolism which further triggered the degradation process of sludge. Therefore, biotransformation of pretreated paper mill sludge by TDS9 was evaluated under optimized culture condition. The optimal conditions for the batch culture of strain TDS9 were determined using CMC as the sole carbon source where 1.25% beef extract was an inducer (nitrogen source) for maximum sugar production. In addition to 1.25% beef extract, 35^o^C and pH 8 are the optimal incubation temperature and initial pH of the culture, respectively.

**Fig. 8:**
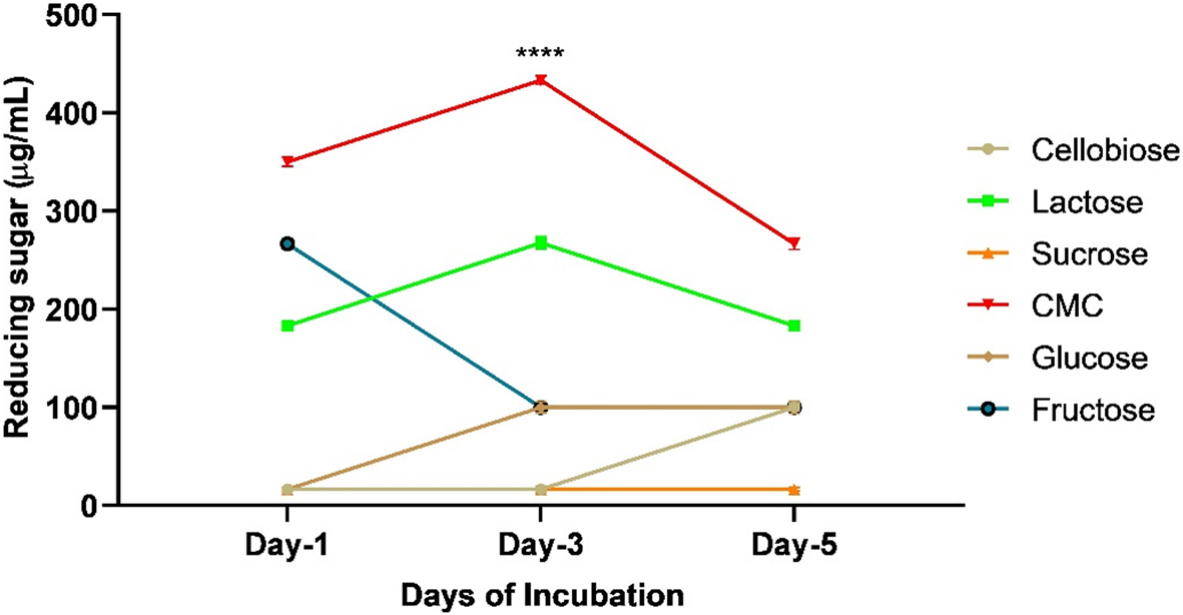
Effect of inducers on paper sludge degradation. The production of reducing sugar in the presence of different types of carbon source are indicated by a separate colored line. The culture media was inoculated with our bacterial isolate *Arthrobacter woluwensis* TDS9 and incubated at 25°C and 150rpm over a range of incubation period of 1–5 days. The initial pH of culture media was set at 8, and the medium contained 1.25% beef extract, 1% paper mill sludge, and 0.15% inducer as co-substrate. ^****^ indicates statistical significance value, *P* < 0.0001

After chemical treatment, the paper sludge was subjected to microbial degradation in the presence of different carbon sources as the co-substrate for cellulase enzyme production. Therefore, in case of paper mill sludge degradation, the MS medium was supplemented with 1% pre-treated paper mill sludge, 1.25% beef extract, and different inducers at 0.15% concentration. Concentration of reducing sugar in broth culture was determined at 24h interval up to 120 h. The effect of inducers on paper mill sludge degradation is presented in Fig. 8. The highest paper mill sludge degradation was observed in the presence of 0.15% CMC, yielding 433.33 μg/mL reducing sugar using the strain TDS9 under optimized culture condition (Fig. 8). Consequently, the amount 433.33 μg/mL reducing sugar obtained from bioconversion of paper mill sludge is the highest amount until today (Fig. 8).

## Discussion

Cellulolytic microorganisms have long been a topic of interest to the scientists for their feasibility to be exploited and numerous industrial applications [58, 59]. The current enzyme repertoire is still inadequate to meet the industrial requirement, and the quest is being continued to isolate cellulolytic strains from unexplored environments [60, 61]. Several cellulase-producing bacterial isolates were isolated from different soil samples and paper mill waste. After screening for qualitative and quantitative cellulase activities, a potential cellulase-producing bacterial isolate TDS9 was finally selected for this study. The 16S rRNA gene sequencing and phylogenetic analysis of the gene sequences confirmed the identity of the isolate TDS9 as *Arthrobacter woluwensis* TDS9 strain. Moreover, morphological and biochemical study of the isolate also aided to characterize the isolate. Although the saccharification of cellulose production results in different simpler sugar forms is a key step of the bioconversion, priority was given to assay the reducing sugar production for each parameter.

Being an inducible enzyme, cellulase is prone to be exploited by its culture condition, and carbon and nitrogen sources [58, 62]. In the present study, different culture conditions were optimized for maximum saccharification of lignocellulosic biomasses. The newly isolated strain *Arthrobacter woluwensis* TDS9 produced the highest amount of reducing sugar from CMC after 72h of incubation which is decreased afterwards. It might be due to the depletion of nutrients in the batch culture medium which may have stressed the bacterial physiology to utilize complex carbon source. Similar incubation period was also reported on the production of cellulase by *Bacillus subtilis* Q3 and *Enhydrobacter* sp. ACCA2 strains [63, 64]. Thus, the production of maximum cellulase at early stage (72h incubation) of fermentation indicated that the strain TDS9 can be useful for large-scale cellulase production.

Therefore, it was observed that the optimum temperature for maximum cellulase production by *A. woluwensis* TDS9 was 25°C which also coincides with the mesophilic nature of the bacterial candidate. Moreover, the production of reducing sugar gradually depleted with the increase of incubation temperature which might be due to the alteration of cell membrane composition and stimulation of protein catabolism at higher temperature. Similar optimum temperature was found for the production of cellulase and xylanase using *Micrococcus luteus* SAMRC-UFH3 KU171371 [65], *Pleurotus sapidas* [66], *Trichoderma viride* VKF3 [67], and for *Bacillus* sp. [68].

Also, the highest cellulase activity at pH 8 indicates slightly alkaliphilic nature of the strain TDS9. Similar optimum initial medium pH was also found for *Thermomonospora curvata* [69] and for *Paenibacillus terrae* [70]. However, the strain *Bacillus subtilis* Q3 also produced maximum cellulase at pH 7 [63]. Premalatha et al. [64] observed the pH 6.5 as optimum medium pH for maximum enzyme production using *Enhydrobacter* sp ACCA2 strain.

Thereafter, among different types of carbon sources, *A. woluwensis* TDS9 was able to produce maximum reducing sugar when CMC was provided as sole carbon source. However, it was found that *A. woluwensis* TDS9 produced maximum sugar when medium was supplemented with simpler form of cellulose rather than those of natural origin with complex structures. Similar findings were observed using CMC as the ideal source of carbon for cellulase production by *Bacillus cereus* LAZ 518 [71] and *Enhydrobacter* sp. ACCA2 [64].

The strain *A. woluwensis* TDS9 yielded the highest amount of reducing sugar from CMC when cultured with beef extract as a nitrogen supplement. Moreover, our results revealed that the inorganic sources of nitrogen were found less capable to induce cellulase activity compared to that of organic and complex form of nitrogen sources. Similar results were also reported by other researchers using *Streptomyces viridobrunneus* SCPE-09 [72], *Pseudomonas* sp. [73], *Bacillus pumilus* B20 [74], and *Psychrobacter aquimaris* LBH-10 [75] strains. In our research work, the optimum conditions of the batch culture for *A. woluwensis* TDS9 strain were optimized for the maximum production of reducing sugar. Therefore, under optimized conditions, a significant product yield (1100.09 μg/mL) of reducing sugar was attained by a novel strain *A. woluwensis* TDS9 at an alkaline pH (pH 8). There is no any report on the production of considerable amount of reducing sugar using this strain TDS9.

Concentration variation of media components exerts a significant effect on cellulase activity [76, 77]. Therefore, optimization of individual components of a medium has been considered as a measure to reduce production cost as well as obtain higher enzyme activity. Subsequently, the highest cellulase activity was observed at 50 °C and 60 min of reaction period, indicating the enzyme is thermo-tolerant which signifies its application for industrial use. Optimum enzyme activity at pH 8 gives us insight about formulating the bioconversion process at which it can function best and the alkaline pH 8 indicates the optimum cellulase activity for hydrolyzing cellulosic substrate by this strain TDS9 in this growth condition, which can be supported by a previous study conducted by McDermid et al. [78]. Similar optimum condition for cellulase activity was also reported by Patagundi et al. [79], Giese et al. [80], and Liang et al. [70] for temperature, reaction time, and pH in *Bacillus cereus*, *Trichosporon mycotoxinivorans* UFMG-CLM68, and *Paenibacillus terrae* ME27-1, respectively.

Endoglucanase IV identified from the zymogram analysis of the newly isolated strain *A. woluwensis* TDS9 was characterized to exhibit enzymatic activity on substrates containing beta-1,4- glycosidic bond, such as carboxymethylcellulose (CMC), hydroxyethylcellulose (HEC), and beta-glucan [81]. Thus, it could be involved in the degradation of complex natural cellulosic substrates*. A. woluwensis* TDS9 showed the highest cellulase activity against CMC indicating its substrate specificity according to the nature of the enzyme itself. CMC induced maximum cellulase activity in TDS9, which is comparable to other studies on cellulolytic bacteria. A comparison of cellulase activity among various other bacteria is presented in Table 3, where CMC was used as a substrate.

**Table 3:** Comparison of CMCase enzyme production using CMC as the carbon source

Strain	Source	Substrate	Enzyme activity (U/mL)	Reference
*Arthrobacter woluwensis* TDS9	Soil	CMC	1.04	This study
*Arthrobacter* sp. HPG166	Hindgut of root-feeding larvae *Holotrichia parallela*	CMC	1.411	[82]
*Bacillus subtilis* AS3	Vermi compost	CMC	0.43	[83]
*Enterobacter cloacae* WPL 214	Bovine rumen fluid waste	CMC	0.09	[84]
*Trichoderma harzianum*	Soil	CMC	0.120	[85]
*Halomonas* sp. strain PS47	Soil	CMC	0.138	[86]

The effect of several nitrogen sources and metal ions on the cellulase activity of the selected isolate was not significant. Moreover, tryptone and potassium chloride slightly increased the cellulase activity compared to that of other nitrogen sources and metal ions tested in this study. This nitrogen sources were unresponsiveness to the extracted enzyme as the external nitrogen content. Nitrogenous compounds do not affect the availability of substrate or catalyze reaction of cellulase with relevant substrate in any ways. Among four different surfactants and reductants SDS inhibited the cellulase activity. This might be due to the fact that surfactants tend to decrease the surface tension of aqueous systems, which may alter the properties of liquids such as detergency, emulsification, greasing, and solubilization. Surfactant properties can decrease the nonproductive adsorption of cellulases on lignin, acting as “activators agents” of these enzymes [87].

Lignocellulosic biomasses pose certain challenges against microbial degradation due to its rigid crystalline structure of cellulose as well as hemicellulose and lignin contents. All these structural complexities restrict the availability of cellulose components for microbial degradation. Thus, the chemical treatment of the lignocellulosic biomasses is a prerequisite for microbial degradation. Alkali treatment of the paper mill sludge successfully loosened the cellulose fibers from lignin and hemicellulose as observed under microscope. While other toxic substances present in paper mill sludge were removed via hydrogen peroxide treatment. Finally, the culture of *Arthrobacter woluwensis* TDS9 was used as an inoculum for the bioconversion of chemically pre-treated paper mill sludges. The significant production of reducing sugar from pre-treated paper mill sludge was observed in the presence of small amount of CMC as an inducer. A similar prevalence of crystalline cellulose or CMC-induced higher rate of endoglucanase activity was reported in several other studies [88–91]. The inducers may have significant role on the induction of cellulase gene activation which then further accomplished the degradation of cellulosic content from paper mill sludge. This result depicted that the strain TDS9 has complex enzyme activity with lignocellulolytic effect and saccharification potential. Therefore, our newly isolated strain *A. woluwensis* TDS9 could utilize cellulose from paper mill sludge as a carbon and energy source for their growth and produce significant amount of bioproducts reducing sugar under aerobic condition. Our report indicated that the batch fermentation with initial paper mill sludge 10 g/L (1%) was the optimal concentration for maximum sugar production, which is the highest concentration in batch culture till now (Fig. 8). Still now, there is no any significant report for the bioconversion of paper mill sludge by bacterial candidate. However, a high product yield (433.33 μg/mL) of reducing sugar obtained from batch culture process was the highest amount using paper mill sludge as a cellulosic feed stock until now. We have proved, however, that a newly isolated bacterial strain *A. woluwensis* TDS9 could utilize paper mill sludge to produce a significant amount of reducing sugar. The primary objective of this study was to obtain simpler carbohydrate form from complex negative cost biomass paper mill sludge. This simpler sugar forms can further be converted into other biofuels or related compounds which have economic significance.

Nevertheless, cellulases have a potential application for hydrolyzing cellulosic biomass in biorefining industries which are based on agro-industrial wastes. The bacterial strain *Arthrobacter woluwensis* TDS9 reported in this study was capable of utilizing lignocellulose substrates including paper mill sludge. This work acts as a step towards for further study on bio-refinery feedstocks that are composed of low or negative cost biomass.

## Conclusions

A potential cellulase-producing bacterial strain isolated from the soil was characterized and identified as *Arthrobacter woluwensis* TDS9. This efficient cellulase producing bacterial strain TDS9 showed a potential activity on the biotransformation of cellulosic biomass as well as primary sludge of paper mill to produce value-added product (reducing sugar). Moreover, finding an eco-friendly method for negative-cost biomass conversion remains an important goal in bioremediation, and implementing microorganisms as biocatalyst is an expressively promising greener method. Therefore, an increased (433.33 μg/mL) sugar production was attained from negative-cost biomass (paper mill sludge) using the newly isolated strain *A. woluwensis* TDS9. It is demonstrated that the strain *A. woluwensis* TDS9 able to utilize paper mill waste (primary sludge) derived from pulp production process has a high cellulose utilization rate and high product yield of reducing sugar. Therefore, further studies with our newly isolated strain TDS9 are granted to boost the utilization rate of cellulosic biomass and the production of reducing sugar. Our result also showed that it is possible to discover novel microbial strains to produce value-added bioproducts using negative cost biomass paper mill sludge. The present study also introduces the novel strain *A. woluwensis* TDS9 as a potential biocatalyst for the saccharification of cellulolytic biomass, which represent the economic significance for biomass bioconversion industries. Thus, the bacterial strain TDS9 could be a potential candidate for cellulase enzyme production.

## Data Availability

All data generated or analyzed during this study are included in this article.

## Abbreviations

CMC: Carboxy methyl cellulose
CMCase: Carboxy methyl cellulase
MS: Minimum salt
LB: Luria-Bertani
rpm: Rotation per minute
DNS: Dinitrosalicylic acid
OD: Optical density
IUPAC: International Union of Pure and Applied Chemistry
mM: Mili molar
SDS: Sodium dodecyl sulfate
EDTA: Ethylenediamine tetraacetic acid
KDa: Kilo Dalton
